# The impact of lifestyle, measured with the HLPCQ questionnaire on the prevalence of metabolic syndrome in Poland: a multicenter study

**DOI:** 10.1038/s41598-024-60866-1

**Published:** 2024-05-02

**Authors:** Mateusz Babicki, Karolina Kłoda, Justyna Ledwoch, Sandra Janiak, Filip Krzyżanowski, Tomasz Zieliński, Patrycja Grabska, Dominik Gajowiak, Wojciech Malchrzak, Agnieszka Mastalerz-Migas

**Affiliations:** 1 Department of Family Medicine, Wroclaw Medical University, 50-367 Wrocław, Poland; 2 MEDFIT Karolina Kłoda, Ul. Narutowicza 13E/11, 70-240 Szczecin, Poland; 3 NZOZ Kraków Południe, Kraków, Poland; 4 Department of Family Medicine, Nicolaus Copernicus University in Torun, Collegium Medicum in Bydgoszcz, 85-094 Bydgoszcz, Poland; 5 Centrum Medyczne AD-MED, Wrocław, Poland; 6 NZOZ PROMED A. Szendała, T. Zieliński – Lekarze sp. p., Wysokie, Poland; 7 Przychodnia Lekarska Rodzina Jerzy Rajewski Sp. J, Koronowo, Poland; 8 Przychodnia Lekarska Michałek, Rychwał, Poland; 9 Department of Family Medicine, Wroclaw Medical University, Ul. Syrokomli 1, 51-141 Wroclaw, Poland

**Keywords:** Obesity, Metabolic syndrome, Life style, Epidemiology of the metabolic syndrome, HLPCQ, Cardiology, Health care, Risk factors

## Abstract

Metabolic syndrome is one of the most common health problems for people around the world. The aim of our study was to assess the prevalence of metabolic syndrome among adults without prior diagnosis of cardiovascular disease, diabetes, and chronic kidney disease. We also plan to assess the influence of certain lifestyle components on prevalence of metabolic syndrome. The study involved cardiovascularly healthy patients undergoing lab tests, measurements, and the HLPCQ questionnaire (The Healthy Lifestyle and Personal Control Questionnaire). The data were used to diagnose metabolic syndrome. Out of 1044 patients from 10 primary care facilities, 23.3% met the metabolic syndrome criteria, showing a strong link with increased blood pressure, cholesterol, and fasting glucose. Lower scores in the Organized physical exercise subscale of the HLPCQ questionnaire were noted in those with metabolic syndrome. Comparing the subscale of HLPCQ questionnaire, the lower results in Organized physical exercise subscale were found among the participants with metabolic syndrome, both male and females. Metabolic syndrome, a significant risk factor for cardiovascular disease, should be screened for actively, even in apparently healthy populations. Results obtained in our study from analysis of HLPCQ show that screening for metabolic syndrome should be preceded by prevention based on regular physical activity and proper eating habits.

## Introduction

Metabolic syndrome is defined as a co-occurrence of certain cardiovascular risk factors which contribute to major health and economic burden worldwide, including Poland^1^.

The concept of metabolic syndrome was introduced over 100 years ago. In 1923 the concomitance of hypertension and hyperglycaemia was reported. 20 years later the connection between abdominal obesity and type 2 diabetes was found. The first definition of metabolic syndrome was proposed by WHO (WHO—World Health Organisation) in 1988^2^. According to this definition the main diagnostic criterium of metabolic syndrome was impaired glucose metabolism, especially insulin resistance. The additional factors were: obesity, dyslipidaemia, elevated blood pressure, microalbuminuria (the presence of at least 2 out of 4 criteria)^3,4^. Despite the progression in understanding of metabolic syndrome pathophysiology, there are still several definitions in use which may lead to some difficulties in comparing data regarding this condition. However some consensus also can be found. The definition proposed by several Polish medical associations describes metabolic syndrome as a presence of obesity (the baseline criterium) defined as waist circumference ≥ 88 cm for females and ≥ 102 cm for males and/or body mass index (BMI) ≥ 30 kg/m2 and at least 2 out of 3 factors: elevated blood pressure, impaired glucose metabolism or elevated non- HDL serum cholesterol level^1^.

The components of metabolic syndrome are all modifiable factors which are also well established cardiovascular disease risk factors. The data from observational studies shows an increase in prevalence of metabolic syndrome among adults in Poland. Those data are consistent with classification of Poland as a high risk country according to ESC 2021^5^.

In 2014 the prevalence of metabolic syndrome in Poland was 33% in females and 39% in males. The increased prevalence of metabolic syndrome was observed in males aged 60–74 years old^6^.

Those data seemed to be consistent with studies from other European countries. In MORGAM study (Monica, Risk, Genetics, Archiving and Monograph) among 69,094 males and females aged 19–78 years old from 10 European countries the prevalence of metabolic syndrome was 22.3% in females and 18.2% in males^7^.

The study conducted in 2007 in Italy with 4418 participants showed prevalence of metabolic syndrome in 33% of patients. Similar study on French population presented incidence of metabolic syndrome at 16% in male population and 11% in females^8^.

In the United States, according to a NHANES study conducted between 2011 and 2012 with 4418 participants, the prevalence of metabolic syndrome was 34.7%. The positive correlation in incidence of metabolic syndrome and age was established with obesity as the most common factor (53%)^9^.

Metabolic syndrome was often identified as an adult-life condition. Due to increased prevalence of obesity among the paediatric population, metabolic syndrome is also an important health issue among this age group^10^.

The causes of metabolic syndrome are multifactorial, combining environmental and genetic factors. The leading contributing factors are: negative change in dietary habits (calories overload, increased consumption of high processed foods) and low levels of physical activity. For example, a study of 6114 women found that a sedentary lifestyle and poor eating habits contributed to an increase in abdominal obesity and abnormal fasting blood glucose^11^. The negative impact of unhealthy eating habits was also confirmed by a study among Iranian women^12^. In contrast, a study among adults living in Korea showed that physical inactivity has a negative impact on the development of metabolic syndrome among both men and women^13^. Some studies showed a correlation between low birth mass and higher incidence of metabolic syndrome^14^. The patient with metabolic syndrome can be easily assessed in primary care settings with anthropometric measurements and laboratory tests. The prevention programs aimed to identify cardiovascular risk factors can also be used to diagnose metabolic syndrome. Currently, in Poland two such prevention programs are available. First one, the cardiovascular disease prevention program called ChUK screens the population between 35 and 65 years old without previously identified cardiovascular disease, diabetes, chronic kidney disease and familial hypercholesterolemia. The assessment of cardiovascular risk profile may be performed by primary care physician or primary care nurse. The data obtained in this prevention program include the following:anthropometric measurements, blood pressure measurement, family history of cardiovascular events, smoking habits, level of physical activity and serum glucose and lipids levels^15^.

The second prevention program is “40 + prophylaxis” and is aimed at the whole population over 40 years old. It consists of a panel of laboratory test and anthropometric measurements. Results from this program can be also used to assess cardiovascular risk factors and metabolic syndrome components^16^.

Despite the contrary data on the positive impact of prevention programs on reducing morbidity and mortality of cardiovascular diseases, it seems they may be helpful in identifying cardiovascular risk factors^17–20^.

Metabolic syndrome is a major health issue also in the younger so called healthy population. It can lead to development of many chronic conditions and increases cardiovascular risk. Interventions contributing reducing metabolic syndrome components as well as cardiovascular risk factors may impact the prevalence of many chronic concomitant conditions (i.e. hypertension, diabetes)^1^.

The aim of our study was to assess the prevalence of metabolic syndrome among adults without prior diagnosis of cardiovascular disease, diabetes, and chronic kidney disease. We also plan to assess the association of certain lifestyle components (dietary choices, physical activity, social and mental balance) on prevalence of metabolic syndrome, according to gender.

## Materials and methods

Our study was designed as a multicentred, retrospective observational study. It was conducted on the Polish population ≥ 35 y.o. without prior diagnosis of CVD, diabetes and chronic kidney disease, who attended the primary care settings to participate in the prevention program.

The inclusion criteria were as follows:

1. Lack of CVD, diabetes and chronic kidney disease when entering the study
2. Age 35–65 y.o.
3. Written consent to participate in the study
4. Performed laboratory tests within 4 weeks before or after entering the study: blood lipids panel (serum total cholesterol, HDL, LDL, non-HDL, triglycerides) and serum fasting glucose level

Patient needed to meet all of 4 inclusion criteria.

The data collection period was between 09.2022 and 05.2023. There were 10 primary care facilities involved in the recruitment process and the data collection. The four of them were located in rural areas and six of them in the cities.

Patients during primary care physician appointment underwent an analysis of medical data: laboratory test (blood lipids panel: serum total cholesterol, HDL, LDL, non-HDL, triglycerides and serum fasting glucose level), age, sex, dwelling place, educational status and professional activity. The information about family history (mother and father) of myocardial infarction and stroke, including the age of disease presentation was collected. Participants were also asked about smoking habits (current smoking, smoking in the past and passive smoking) and levels of physical activity. In the area of physical activity we assessed the intensity and the duration (minutes per week). The medium level of physical activity was defined by activity that causes faster breathing and faster heart rate (i.e. riding a bike at normal pace, playing volleyball or fast walking). The intense physical activity was described as an activity that causes very fast breathing and very fast heart rate (i.e. heavy lifting, aerobic exercises, running, riding a bike at faster pace). The anthropometric measurements were performed (height and weight with BMI calculation, waist circumference) as well as heart rate and blood pressure.

The waist circumference was measured in the point of a half-distance between costal arch and the highest point of iliac crest in the mid-axillary line as stated in the recommendations^1^.

In the last step, the participants were asked to fill the HLPCQ questionnaire. It was firstly introduced in Greece and in 2021 was validated and adapted for the Polish population. The HLPCQ was designed as a 26 question tool to assess the frequency of some lifestyle habits. The stratification was based on Likert 4 degree scale: 1- never or rarely, 2- sometimes; 3—often; 4—always. From 26 questions—12 address dietary choices and habits and 2 evaluate the level of physical activity. There are 4 questions which assess social factors and mental balance and 8 questions in the area of daily routine and time management.

In addition to the total score analysis, there is possibility to evaluate the scores in 5 subscales:

1) Healthy dietary choices—questions—1,3,4,5,13,14,16—maximum score—28.

2) Dietary harm avoidance—questions—8,9,10,11—maximum score—16.

3) Daily routine—questions—2,6,7,12,15,17,19,22—maximum score—32.

4) Organized physical exercise—questions—20,23—maximum score—8.

5) Social and mental balance—questions—18, 21,24, ok 25,26—maximum score—20.

The evaluation of the questionnaire is based on total score. The higher number of points correlates with a healthier lifestyle. The maximum score is 104. The assessment in each subscale is also possible^21^.

HLPCQ tool has high alfa Cronbach internal consistency-0.898.

### Metabolic syndrome

The diagnosis of metabolic syndrome was made if obesity (defined by BMI) or abdominal obesity was present and 2 out of 3 additional criteria were fulfilled:

- serum fasting glucose ≥ 100 mg/dl
- serum non-HDL ≥ 130 mg/dl
- blood pressure ≥ 130 and/or 85 mmHg

This definition of metabolic syndrome was proposed by the panel of experts in 2022. According to those recommendations obesity was defined by BMI ≥ 30 and abdominal obesity by waist circumference ≥ 88 cm in females and ≥ 102 in males.^1^

### Statistical analysis

The analysed data had quantitative and qualitative characteristics. We used the Shapiro- Wilk test. The assessment of qualitative data was made by the chi 2 test. To analyse the quantitative parameters we used Mann–Whitney U test. Statistical significance was assumed at the level of < 0.05. Subsequently, a complex logistic regression model was built, where the dependent variable was the diagnosis of metabolic syndrome, and the independent variables included gender, age, place of residence by the scores of the HLCPQ scale and its individual subscales. Calculations were performed using Statistical 13 software by TIBCO Software Inc. (Palo Alto, CA, USA).

### Informed consent statement

All participants were fully informed about the aims of the study and signed the informed consent form prior to completing the research instruments. Participation was voluntary, and confidentiality and anonymity were safeguarded at all times.

## Results

### Characteristic of the research group

1044 patients from 10 primary care facilities were enrolled in the study after meeting the inclusion criteria. Median of age was 47.9 ± 9.3 years old The predominance of female gender (64,4%) was found. 1 out of 4 participants were current smokers and 16.9% of the studied group declared smoking in the past. The metabolic syndrome criteria was met by 242 participants (23.3%). The prevalence of metabolic syndrome was higher in rural areas and in participants with low levels of physical activity. Patients with higher educational status and white-collar workers presented with metabolic syndrome less often.

Detailed description is presented in Table 1.

**Table 1:** Characteristics of the study group, including the division into patients with and without diagnosed metabolic syndrome.

Variable	The whole group N (%)	Metabolic syndrome N (%)	Lack of metabolic syndrome N (%)	p
Age M ± SD	47.9 ± 9.3	46.7 ± 8.9	51.9 ± 9.1	** < 0.001†**
Sex				
Female	672 (64.4)	150 (22.3)	522 (77.7)	0.376‡
Male	372 (35.6)	92 (24.7)	280 (75.3)	
Type of profession				
White-collar worker	464 (44.4)	75 (16.2)	389 (83.8)	** < 0.001‡**
Manual worker	290 (27.8)	60 (20.7)	230 (79.3)	
Farmer	85 (8.1)	34 (40.0)	51 (60.0)	
Pensioner	100 (9.6)	41 (41.0)	59 (59.0)	
Other	105 (10.1)	32 (30.7)	73 (69.3)	
Education				
Basic vocational	231 (22.1)	76 (36.8)	155 (67.2)	** < 0.001‡**
Secondary	286 (27.4)	64 (22.4)	222 (77.6)	
Higher	462 (44.3)	79 (17.1)	383 (82.98)	
Other	65 (6.2)	23 (35.4)	42 (64.6)	
Place of residence				
Village	326 (31.2)	104 (31.9)	222 (68.1)	** < 0.001‡**
City of up to 50 000 inhabitants	261 (25.0)	68 (26.1)	193 (73.9)	
City of over 50 000 inhabitants	457 (43.8)	70 (15.3)	387 (84.7)	
Heart attack and/or stroke in a father under 55?	91 (8.7)	23 (25.3)	68 (74.7)	0.621‡
Heart attack and/or stroke in a mother under 65?	48 (4.6)	8 (16.7)	40 (83.3)	0.277‡
Smoking				
Smokes cigarettes	248 (23.8)	71 (28.6)	177 (71.4)	0.112‡
Smoked in the past	177 (16.9)	41 (23.2)	136 (76.8)	
Passive smoking	22 (2.1)	4 (18.2)	18 (81.8)	
Does not smoke	597 (57.2)	126 (21.1)	471 (78.9)	
Moderate physical activity				
I don't do that kind of activity	413 (39.6)	113 (27.4)	300 (72.6)	**0.048‡**
1–2 times a week	211 (20.2)	43 (20.4)	168 (79.6)	
3–4 times a week	188 (18.0)	35 (18.6)	153 (81.4)	
> 4 times a week	232 (22.2)	51 (21.9)	181 (78.1)	
Average time of moderate physical activity [min/week]	189.2 ± 420.5	221.6 ± 488.7	179.4 ± 397.4	0.151†
Intense physical activity				
I don't do that kind of activity	816 (78.2)	199 (24.4)	617 (75.6)	**0.043‡**
1–2 times a week	96 (9.1)	21 (21.9)	75 (78.1)	
3–4 times a week	73 (7.0)	12 (16.4)	61 (83.6)	
> 4 times a week	59 (5.7)	10 (16.9)	49 (83.1)	
Average time of intense physical activity [min/week]	37.3 ± 116.8	25.5 ± 83.1	40.9 ± 124.9	**0.047†**

p—statistical significance; M—mean; SD—standard deviation; N—number; †Mann–Whitney U test, ‡Chi-squared test; statistically significant values are in bold with the significance level set at p < 0.05.

### Biochemical test analysis

Comparison analysis showed positive association with presence of metabolic syndrome (both in male and females) and higher systolic, diastolic blood pressure and higher serum total cholesterol, LDL, non-HDL, triglycerides and fasting glucose. The negative association was found with HDL cholesterol. Detailed description is presented in Tables 2 and 3.

**Table 2:** Comparison of laboratory test results, weight, height, BMI and waist circumference between patients with and without diagnosed metabolic syndrome among men.

Variable	The whole group (N = 372)	Metabolic syndrome (N = 92)	Lack of metabolic syndrome (N = 280)	p
Weight [kg]	87.7 ± 15.3	101.4 ± 16.9	93.1 ± 9.6	** < 0.001†**
Hight [cm]	177.4 ± 10.8	175.9 ± 17.9	177.8 ± 7.0	0.479†
BMI	27.8 ± 4.4	32.3 ± 4.3	26.3 ± 3.3	** < 0.001†**
BMI ≥ 30	93 (24.2)	65 (69.9)	28 (30.1)	** < 0.001‡**
Waist circumference [cm]	97.4 ± 12.3	110.5 ± 10.1	93.1 ± 9.6	** < 0.001†**
Abdominal obesity N(%)	121 (32.5)	81 (66.9)	40 (33.1)	** < 0.001‡**
SBP [mmHg] M ± SD	134.1 ± 16.8	144.2 ± 17.4	130.8 ± 15.4	** < 0.001†**
DBP [mmHg] M ± SD	83.6 ± 10.4	90.2 ± 10.1	81.5 ± 9.6	** < 0.001†**
Heart action [beats per minute]M ± SD	73.3 ± 10.7	74.6 ± 11.6	72.8 ± 10.3	0.407†
Total cholesterol [mg/dl] M ± SD	203.3 ± 39.0	210.6 ± 40.1	200.9 ± 38.4	**0.016†**
LDL [mg/dl] M ± SD	127.3 ± 34.3	133.6 ± 35.8	125.2 ± 33.6	**0.009†**
HDL [mg/dl] M ± SD	52.5 ± 13.2	49.3 ± 12.7	53.6 ± 13.2	**0.007†**
Non-HDL [mg/dl]M ± SD	150.5 ± 37.8	162.0 ± 38.0	146.7 ± 37.3	** < 0.001†**
Triglycerides [mg/dl] M ± SD	128.2 ± 69.5	156.9 ± 79.2	118.7 ± 63.9	** < 0.001†**
Glucose [mg/dl]M ± SD	96.1 ± 17.9	101.5 ± 23.4	94.3 ± 15.4	** < 0.001†**

BMI—body mass index, kg—kilograms, p—statistical significance; M—mean; SD—standard deviation; N—number; mmHg—millimetres of mercury; mg/dl—milligrams per decilitre; SBP—Systolic Blood Pressure; DBP—Diastolic Blood Pressure, HR—Heart Rate, LDL—low-density lipoprotein; HDL—high-density lipoprotein †Mann–Whitney U test; ‡Chi-squared test; statistically significant values are in bold with the significance level set at p < 0.05.

**Table 3:** Comparison of laboratory test results, weight, height, BMI and waist circumference between patients with and without diagnosed metabolic syndrome among women.

Variable	The whole group (N = 672)	Metabolic syndrome (N = 150)	Lack of metabolic syndrome (N = 522)	p
Weight [kg]	70.4 ± 15.3	85.8 ± 16.1	66.0 ± 11.8	** < 0.001†**
Hight [cm]	164.1 ± 6.4	163.7 ± 6.4	164.1 ± 6.4	0.543†
BMI	26.1 ± 5.5	31.9 ± 5.4	24.5 ± 4.2	** < 0.001†**
BMI ≥ 30	139 (20.6)	90 (64.8)	49 (35.2)	** < 0.001‡**
Waist circumference [cm]	84.5 ± 13.1	99.5 ± 10.6	80.2 ± 10.3	** < 0.001†**
Abdominal obesity N(%)	247 (36.8)	146 (59.1)	101 (40.9)	** < 0.001‡**
SBP [mmHg] M ± SD	126.8 ± 17.0	142.3 ± 15.3	122.3 ± 14.7	** < 0.001†**
DBP [mmHg] M ± SD	80.4 ± 9.8	88.1 ± 9.3	78.1 ± 8.7	** < 0.001†**
Heart action [beats per minute]M ± SD	75.1 ± 10.7	76.4 ± 11.4	74.7 ± 10.5	0.198†
Total cholesterol [mg/dl] M ± SD	205.0 ± 40.8	224.2 ± 36.2	199.5 ± 40.4	** < 0.001†**
LDL [mg/dl] M ± SD	122.7 ± 38.0	143.1 ± 35.3	116.8 ± 36.7	** < 0.001†**
HDL [mg/dl] M ± SD	62.9 ± 15.7	55.2 ± 12.7	65.1 ± 15.8	** < 0.001†**
Non-HDL [mg/dl]M ± SD	142.5 ± 41.6	169.9 ± 35.4	134.6 ± 39.8	** < 0.001†**
Triglycerides [mg/dl] M ± SD	108.0 ± 60.2	140.8 ± 58.9	98.5 ± 57.3	** < 0.001†**
Glucose [mg/dl]M ± SD	92.1 ± 16.0	101.9 ± 27.8	89.3 ± 8.5	** < 0.001†**

BMI—body mass index, kg—kilograms, p—statistical significance; M—mean; SD—standard deviation; N—number; mmHg—millimetres of mercury; mg/dl—milligrams per decilitre; SBP—Systolic Blood Pressure; DBP—Diastolic Blood Pressure, HR—Heart Rate, LDL—low-density lipoprotein; HDL—high-density lipoprotein †Mann–Whitney U test; ‡Chi-squared test; statistically significant values are in bold with the significance level set at p < 0.05.

### Selected lifestyle parameters.

Comparing the subscale of HLPCQ questionnaire, the lower results in Organized physical exercise subscale were found among the participants with metabolic syndrome, both male and females. Females with metabolic syndrome presented with lower results in Healthy dietary choices subscales. In the male population the difference was found in Dietary harm avoidance subscales. Detailed description of results from subscales is presented in Tables 4 and 5. A complex logistic regression model showed that among the lifestyle parameters analyzed, after accounting for the influence of age and place of residence, regular physical activity has a significant impact on reducing the risk of developing metabolic syndrome among both men and women. The exact results of the regression analysis are shown in Table 6.

**Table 4:** Summary of HLPCQ scale results for the entire group and for people with and without metabolic syndrome among men.

HLPCQ	The whole group M ± SD	Metabolic syndrome M ± SD	Lack of metabolic syndrome M ± SD	p
Healthy dietary choices	15.2 ± 3.9	15.0 ± 3.5	15.3 ± 4.0	0.768
Dietary harm avoidance	10.4 ± 2.7	9.8 ± 2.7	10.6 ± 2.6	**0.012**
Daily routine	19.8 ± 5.8	18.8 ± 5.4	20.2 ± 5.9	0.067
Organized physical exercise	4.0 ± 1.8	3.5 ± 1.6	4.2 ± 1.8	**0.002**
Social and mental balance	12.3 ± 3.0	11.9 ± 3.0	12.4 ± 3.0	0.145
Final result	61.8 ± 13.2	59.1 ± 13.5	62.7 ± 13.5	**0.039**

M—mean; SD—standard deviation; Mann–Whitney U Test statistically significant values are in bold with the significance level set at p < 0.05.

**Table 5:** Summary of HLPCQ scale results for the entire group and for people with and without metabolic syndrome among women.

HLPCQ	The whole group M ± SD	Metabolic syndrome M ± SD	Lack of metabolic syndrome M ± SD	p
Healthy dietary choices	18.0 ± 3.6	17.5 ± 3.4	18.2 ± 3.6	**0.047**
Dietary harm avoidance	11.3 ± 2.8	11.2 ± 2.8	11.3 ± 2.9	0.731
Daily routine	20.8 ± 5.4	20.5 ± 5.4	21.0 ± 5.4	0.195
Organized physical exercise	4.3 ± 1.8	3.9 ± 1.6	4.3 ± 3.9	**0.025**
Social and mental balance	13.1 ± 3.0	13.2 ± 3.0	13.1 ± 3.0	0.956
Final result	67.6 ± 12.4	66.4 ± 12.0	67.9 ± 12.5	0.157

M—mean; SD—standard deviation; Mann–Whitney U Test statistically significant values are in bold with the significance level set at p < 0.05.

**Table 6:** Results of a complex logistic regression model assessing the effect of age, place of residence and individual lifestyle parameters on the risk of developing metabolic syndrome for women and men.

Variables	Female		Male	
	OR [95Cl]	p	OR [95Cl]	p
Age, years	1.08 [1.05, 1.10]	** < 0.001**	1.02 [0.99, 1.06]	0.064
Place of residence, City of up to 50 000 inhabitants	0.67 [0.41, 1.09]	0.111	0.89 [0.47, 1.67]	0.714
Place of residence, City of over 50 000 inhabitants	0.37 [0.23, 0.59]	** < 0.001**	0.88 [0.49, 1.62]	0.681
Healthy dietary choices	0.96 [0.89, 1.03]	0.341	**0.88 [0.78, 0.94]**	**0.011**
Dietary harm avoidance	0.99 [0.92, 1.07]	0.941	**0.85 [0.75, 0.95]**	**0.005**
Daily routine	0.97 [0.92, 1.02]	0.239	0.97 [0.92, 1.02]	0.264
Organized physical exercise	0.90 [0.78, 0.99]	**0.043**	**0.81 [0.69, 0.97]**	**0.019**
Social and mental balance	1.07 [0.99, 1.17]	0.070	0.98 [0.89, 1.10]	0.839
Final result	0.97 [0.95, 1.09]	0.145	0.98 [0.97, 1.3]	0.231

OR—odds ratio; CI—confidence interval, significant values are in bold with the significance level set at p < 0.05.

## Discussion

In this study we hypothesized that Healthy Dietary Choices, Dietary Harm Avoidance, Daily Routine, Organized Physical Exercise and Social and Mental Balance are associated with the metabolic syndrome occurrence and that this association might differ between males and females. We have found 23.2% prevalence of metabolic syndrome among the population considered as healthy and thus, participating in CVD prevention program. Occurrence of metabolic syndrome was higher among males than females (24.7% vs. 22.3%) and was associated with demographic characteristics of studied participants. We have observed an association between lower level of Organized Physical Exercise with metabolic syndrome in both sexes, lower level of Healthy Dietary Choices in females as well as Dietary Harm Avoidance in males with metabolic syndrome.

The prevalence of metabolic syndrome varies between geographical regions (sometimes even on provincial levels), genders and ethnic groups^22–25^. Economic and occupational status are also factors contributing to those differences. The heterogeneity of metabolic syndrome criteria may also influence the statistics^26,27^. Most of the studies are consistent on association between age and increased prevalence of metabolic syndrome correlation ^25,28–31^. The global prevalence of metabolic syndrome varies from 12.5% to 31.4% based on the definition. It seems to be significantly higher in the Eastern Mediterranean Region and Americas and with the country's level of income^32^. However in the study assessing the metabolic syndrome among children and adolescents, the prevalence of metabolic syndrome was not consistently higher with increasing levels of development^33^. On the other hand in the low income countries association between urbanization and increased prevalence of metabolic syndrome was found^34^. Moreover, the differences in prevalence of metabolic syndrome may vary between urbanized and rural areas within the same region. In the meta-analysis of 35 studies from China, the population living in rural areas had a lower prevalence of metabolic syndrome compared to the urban areas 31.3%). However, data from a Bangladesh study which was conducted mostly in rural areas showed the pooled prevalence of metabolic syndrome at 30%^26^. In view of our study Poland can be placed in the middle of metabolic syndrome prevalence ranges (23.2%) with a higher incidence in residents of rural areas, farmers and individuals with lower levels of education. Contrary to the results of our study, most of the studies show the tendency of higher prevalence of metabolic syndrome in females^23,27–29,35,36.^ Some studies showed no statistically significant difference between genders^26,30,31^.

Increased body weight and large waist circumference are major risk factors for metabolic syndrome^37^. Physical inactivity was found to be a significant risk factor for metabolic syndrome^38–40^. This has been confirmed by numerous studies. For example, a study of postmenopausal women found that physical inactivity is one of the most significant factors that increase the risk of developing metabolic syndrome^11^. In contrast, a study on a group of 532 Chinese women found that an unhealthy lifestyle has a significant impact on the risk of developing metabolic syndrome more strongly expressed in men than in women^41^. Converging observations were made in a study conducted by M. Al Thani, where poor diet, low physical activity and smoking were associated with a twofold increase in the risk of developing metabolic syndrome^42^. The other contributing factors are smoking, high-fat diet, sugar sweetened beverages^39,43^. We should also mention the impact of sleep on the risk of developing metabolic syndrome. It has been proven that both too little and too much sleep affect the increased risk of weight gain and increase in body fat^44^. Among dietary habits, stimulants used should also be mentioned the negative effect of alcohol on weight gain, triglyceride levels and blood pressure, and thus accelerate the development of metabolic syndrome^45^. Some studies point out that lower educational status may be associated with high prevalence of metabolic syndrome, as well as some occupations such as drivers, firefighters and emergency department services clinicians. These, sometimes contrary epidemiological data enlighten the heterogeneity and complexity of metabolic syndrome and CVD risk factors. The importance of early diagnostic and therapeutic intervention seems to be crucial as metabolic syndrome is associated with a twofold increase in cardiovascular outcomes, 1.5-fold increase in all-cause mortality as well as increased risk of type 2 diabetes^23,35,46^.

In a meta-analysis of prospective cohort studies in the elderly population, metabolic syndrome was associated with an increased risk of all-cause and CVD mortality^47^. The other study stressed that female gender in the older population with metabolic syndrome presents higher risk of CV mortality compared to males^48^. In the 13-year prospective study on Mediterranean population-based cohort metabolic syndrome was associated with higher risk of incidence of major cardiovascular event, cardiovascular and all-cause mortality, but was neither associated with higher risk of myocardial infarction or stroke^49^. Interestingly, single components of the metabolic syndrome were associated with a similar magnitude of increased CVD^49^. In white adults, higher waist circumference was positively associated with higher mortality at all levels of BMI from 20 to 50 kg/m2^50^. There are some indications that metabolic syndrome may be a risk factor for stroke recurrence^51^. The correlation between metabolic syndrome and atrial fibrillation was also found^22^.

Mierzecki et al. conducted a series of studies on young people from Poland (19–39 years old), initially considered as healthy adults. However, after assessment of all CVD risk factors it turned out that the analysed population was burdened with excessive body mass (BMI ≥ 25 kg/m2 was found in 38.7% subjects) or dyslipidaemia (71.7% individuals). Moreover significant metabolic differences between males and females were observed, which convinced the researchers that the division to sexes should be maintained during further analysis^52,53^ These findings are in agreement with our study where older, but still considered until now as healthy population, was characterized with common occurrence of increased waist measurement, arterial hypertension, dyslipidaemia or hyperglycaemia constituting the metabolic syndrome. Another study from Poland conducted by Kalinowska et al. enrolled patients with schizophrenia (mean age 41.89 ± 9.7 years), thus population burdened with a chronic mental disease. The prevalence of metabolic syndrome in this population was 51%. Such high rate is associated not only with the course of the disease (social withdrawal, lower level of physical activity, poor eating pattern), but also with pharmacotherapy including atypical neuroleptics, of which clozapine and olanzapine have the highest influence of metabolic syndrome development^54^. It can be easily seen that the occurrence of metabolic syndrome in the group of Poles with mental illness is twice as high as in the so-called healthy population analysed in our study. This leads to an assumption that initially the Polish population is characterized with presence of risk factors common for cardiovascular disease and metabolic syndrome. Due to such a high burden, preventive programs and interventions focused on a healthy lifestyle seem justified.

To our knowledge there are no studies evaluating the use of HLPCQ in individuals with metabolic syndrome. Moreover, research on linkage between healthy eating patterns, physical activity and interventions designed to decrease the risk of metabolic syndrome development is scarce. One of significance for this topic is a recent, cross-sectional study from China evaluating the personal and treatment control as well as coherence in 275 participants with high risk of metabolic syndrome development. It was shown that these are indeed key determinants of health behaviour, enabling the design of suitable health interventions^55^. Another significant for our study report, originating from South Korea, scoped on the topic of health promotion behaviours among working adults at risk for metabolic syndrome, which occurrence is quite similar to our studied population (26% vs 23.2%). Five health promotion behaviours were identified: physical exercise, healthy food choices, avoiding fatty foods, eating a nutritious and balanced diet, and eating regular moderate meals. Also in this study, perceived behavioural control was a key predictor or determinant of health behaviours^56^. We have observed that lower levels of physical activity are associated with metabolic syndrome regardless of sex, but in the case of eating pattern—Healthy Dietary Choices were significant for females and Dietary Harm Avoidance for males. The interpretation of this result in our population could be explained by differences in cultural roles of both sexes. The domain of Healthy Dietary Choices in HLPCQ is based on questions regarding active evaluation of quality and quantity of food, calculating calories, checking food labels and preparing food. Thus, the activities that are usually connected to women in Poland. Whereas, the domain of Dietary Harm Avoidance is based on questions regarding omitting fast-foods, sweetened beverages or big portions of food—without requiring active quality assessment or in-depth knowledge about food products.

The authors are aware of the limitation of the present study, which is undoubtedly the lack of representativeness of the study group for Polish society. In addition, there is no data on patients' use of medications other than those used to treat diabetes, cardiovascular disease or chronic kidney disease, which may affect patients' weight. Moreover, our study did not analyse alcohol consumption and sleep assessment, which affect patients' health well-being. Therefore, it is necessary to conduct further studies on a representative group of patients.

### Conclusions

As metabolic syndrome is a common health condition, identified as an important risk factor for CVD, it is necessary to actively search for its components even in populations considered as healthy. The results obtained in our study from the HLPCQ analysis indicate that regular physical activity is an important lifestyle factor for reducing the risk of developing metabolic syndrome in both men and women. The preventive non-pharmacological measures need to be addressed from multiple angles including lifestyle, cultural and socioeconomic aspects and often should depend on regional needs rather than on universal approach.

## Data availability

The data will be available upon contacting the corresponding author.
